# Early Expression of FcγRI (CD64) on Monocytes of Cardiac Surgical Patients and Higher Density of Monocyte Anti-Inflammatory Scavenger CD163 Receptor in “On-Pump” Patients

**DOI:** 10.1155/2008/235461

**Published:** 2008-02-19

**Authors:** Martina Kolackova, Manuela Kudlova, Pavel Kunes, Vladimir Lonsky, Jiri Mandak, Ctirad Andrys, Karolina Jankovicova, Jan Krejsek

**Affiliations:** ^1^Department of Clinical Immunology and Allergy, Medical School in Hradec Králové, Charles University in Prague, Sokolska Street 581, 50005 Hradec Králové, Czech Republic; ^2^Department of Cardiosurgery, Medical School in Hradec Králové, Charles University in Prague, Sokolska Street 581, 50005 Hradec Králové, Czech Republic

## Abstract

*Objective*. Activation of innate immunity cells is inseparably linked to cardiac surgical operation. The aim of this study was to assess the kinetics in the expression of receptor for Fc part of IgG, FcγRI (CD64), and scavenger receptor CD163 on peripheral blood cells of cardiac surgical patients and to examine the effect of cardiac bypass as a separable influence on the systemic acute inflammatory response. *Methods*. Forty patients, twenty in each group, were randomly assigned to CABG surgery performed either with “on-pump” or without “off-pump” cardiopulmonary bypass. Standardized quantitative flow cytometry method was used to determine the expression of surface markers. *Results*. The density of CD64 molecule on monocytes reached maximum on the 1st postoperative day (P<.001) whereas the peak for CD64 molecule expression on granulocytes was postponed to the 3rd postoperative day (P<.001). The expression of CD163 scavenger molecule on monocytes reached maximum on the 1st postoperative day (P<.001). The density of CD163 molecule on monocytes on the 1st postoperative day is significantly higher in “on-pump” patients in comparison with “off-pump” patients (P<.001). *Conclusion*. In cardiac surgical patients the expression of activation marker FcγR1 (CD64) on monocytes is increased earlier in comparison with granulocytes in both “on-pump” and “off-pump” patients. The expression of scavenger molecule CD163 on monocytes is significantly higher in “on-pump” patients.

## 1. INTRODUCTION

Numerous events, potentially generating an inflammatory response, are induced during cardiac surgery. Amongst them, the combination of surgical injury, mechanical manipulation with the heart, the contact of blood components with the artificial surfaces of the cardiopulmonary bypass circuit, transient endotoxemia and ischemia-reperfusion injury of the heart and lungs are relevant [1]. The inflammatory reaction is the result of a complex interplay between numerous humoral factors and cell substrate of inflammation. Amongst cells involved in this process special role is devoted to innate immunity monocyte-macrophages and granulocytes. Whereas monocyte-macrophage cells are the richest source of pluripotent proinflammatory cytokines upon activation, activated granulocytes are recruited into tissues by stepwise interaction between adhesion molecules on the surface of leukocytes and their corresponding receptors expressed on the lumenal surface of inflamed endothelium [2]. There is a substantial long lasting effort to identify activated monocytes and neutrophils in blood of patients with systemic inflammatory response induced by various stimuli either to identify patients at the risk of development of overwhelming inflammatory response potentially ultimating into multiple organ failure syndrome (MOFS) or to implicate the causative agent of such inflammatory response, for example, bacterial infection [3].

Activation of myeloid cells by various physiological and experimental stimuli is accompanied by multiple surface changes associated predominantly with degranulation. Thus, activated blood myeloid cells typically upregulate surface expression of chemotactic receptors, complement receptor type 3 (CD11b/CD18), and downmodulate surface density of lipopolysacharide (LPS) receptor CD14, the low affinity Fc*γ*RIII or CD16 receptor, adhesion receptors CD44 and CD62L [4].

The Fc*γ*-receptor I, Fc*γ*RI, (CD64), is a high affinity receptor for IgG1 and IgG3 subclasses of immunoglobulins. Fc*γ*RI is constitutively expressed with high density on monocytes and macrophages, less so on eosinophils, but only to a very low extent on resting neutrophils.

Numerous substances both exogenous and endogenous origins rapidly upregulate Fc*γ*RI expression on the surface of neutrophils [5]. Microbial cell wall components such as LPS, endogenous complement split products, and cytokines, such as IFN*γ* and TNF*α*, are some of the activators. The expression of Fc*γ*RI is determined by immunofluorescence and flow cytometry. The introduction of a new diagnostic kit Leuko64 enables standardized and quantitative approach to the determination of Fc*γ*RI expression on immune cells.

Hemoglobin scavenger receptor CD163 is a group B cysteine-rich scavenger receptor expressed exclusively by cells of monocyte-macrophage lineage. This glycoprotein is characterized as a scavenger receptor for hemoglobin, mediating endocytosis of hemoglobin-haptoglobin complexes. Previous studies have indirectly linked CD163 scavenger receptor to anti-inflammatory phenomena [6]. High CD163 expression correlates with the Mo2 anti-inflammatory properties of monocytes and macrophages [7].

The aim of this study was to follow the changes in the expression of granulocyte activation markers Fc*γ*RI and anti-inflammatory CD163 scavenger receptor in patients undergoing cardiac surgical operation either with the use of cardiopulmonary bypass (“on-pump”) or operated on the beating heart during “off-pump” operation and in the postoperative period using quantitative flow cytometric approach.

## 2. PATIENTS

Forty patients (31 males, mean age 67.9±9 and 9 females, mean age 66.4±6.4, collective mean age 67.6±8.5 years) referred to first-time coronary artery bypass grafting (CABG) were enrolled in this study. Patients underwent either conventional myocardial revascularization with cardiopulmonary bypass and cardioplegic arrest of the heart (“on-pump,” * n* = 20, 16 males, 4 females, mean age 69.4±7) or beating heart surgery (“off-pump,” * n* =20, 15 males, 5 females, mean age 65.9±9.7).

Patients in both groups were comparable in age, preoperative left ventricular ejection fraction (median 0.65 in “on-pump,” 0.65 in “off-pump” patients, resp.) and the number of performed coronary anastomoses (median 2.0 in “on-pump,” 2.0 in “off-pump,” resp.). The study protocol was approved by the Ethics Committee of the University Hospital in Hradec Králové. All participants were informed in detail about the purpose of the study both orally and in writing. They were free to ask any questions. One person refused to participate for reasons he would not specify. All active subjects have given written informed consents.

Cardiopulmonary bypass, “off-pump” technique, and anesthesiological management have been recently described in detail elsewhere [8].

## 3. BLOOD SAMPLING

Peripheral venous blood from an antebrachial vein was withdrawn in the operating room and in the intensive care unit. Samples were collected into heparinized tubes Vacutainer, Cat. no. 36884 manufactured by Becton Dickinson.

In both “on-pump” and “off-pump” patients, blood was withdrawn at the following time points:

1. introduction to anaesthesia, which in both groups represented the baseline or reference value for all parameters measured thereafter,
2. after termination of the operation,
3. the first postoperative day,
4. the third postoperative day,
5. the seventh postoperative day.

Additional samples were taken from “on-pump” patients:

(1a) before cross-clamping of the aorta,
(1b) after aortic cross-clamp release,
(1c) after termination of CPB.

## 4. MATERIALS AND METHODS

Leuko64 kit manufactured by Trillium Diagnostics, LLC, (www.trilliumdx.com, Brewer, Me, USA) was used to determine the expressions of CD64 and CD163 on leukocytes of blood samples. Leuko64 kit is composed of a reagent cocktail of two monoclonal antibodies with specifities to CD64 (clones 22 and 32.2, both FITC conjugated) and monoclonal antibody to CD163 (clone Mac2-148, phycoerythrin conjugated) and a fluorescence beads suspension used for instrument calibration and standardization of leukocyte CD64 and CD163 expressions in human blood. The assay was run according to the instruction for use provided by manufacturer. Briefly, 50 microliters of blood and the Leuko64 monoclonal cocktail reagent are incubated for 10 minutes, red cell lysis buffer is added and incubated for additional 15 minutes, and 5 microliters of bead suspension are added prior to flow cytometric analysis. Results were measured by an FACSCalibur flow cytometer (BD Biosciences, San Jose, Calif, USA) using CELLQuest software. The listmode data were analyzed using Leuko64 software (Trillium Diag.). Results are expressed as indexes of positivity for CD64 and CD163 expressions on granulocyte and monocyte populations as provided by the Leuko64 software.

## 5. STATISTICAL ANALYSIS

We compared changes in the intensity of expressions of CD64 and CD163 in both groups of patients (“on-pump,” “off-pump”) separately. Samples taken at the introduction to anesthesia were considered as reference or baseline expressions of CD64 and CD163. Differences between “off- and on-pump” patients were also evaluated.

Data were analyzed using two-way ANOVA for repeated measures with Fisher test for multiple comparisons. To exclude confounding effect of different age and sex presentation in both groups, unpaired t-test and chi-square were performed. A probability (*P*) value < .05 was considered significant. Statistical analysis was performed with Statistica 5.5 software (Statsoft, Okla, USA).

## 6. RESULTS

**Figure 1:**
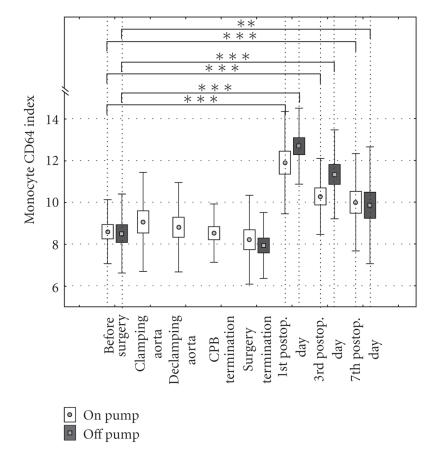
CD64 molecule expression on monocytes in “on-pump” and “off-pump” patients.

**Figure 2:**
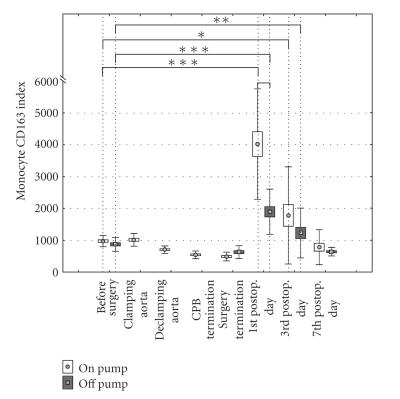
CD163 molecule expression on monocytes in “on-pump” and “off-pump” patients.

We found substantial dynamics in the expressions of both CD64 and CD163 molecules on immune cells in our cardiac surgical patients. These changes are expressed as changes in CD64 and CD163 indexes separately for monocytes and granulocytes. Preoperative levels were taken as reference points. There were no significant changes in the monocyte CD64 expression during cardiac operation in “on-pump” patients (data not shown). The monocytes CD64 index was significantly increased in both “on-pump” and “off-pump” patients in postoperative period from the first to the seventh day (*P* < .01). Comparing monocyte CD64 index between “on-pump” and “off-pump” patients, there were no significant differences (*P* < .587) during operation and in the postoperative period (Figure 1). The similar patterns were found for monocyte CD163 index in our cardiac surgical patients with following exceptions. Monocyte CD163 index, in contrast to the monocyte CD64 index showed a significant difference as a function of the pump status. There was a statistically significant increase in monocyte CD163 index in “on-pump” patients compared to “off-pump” patients at the first postoperative day (*P* < .001) (Figure 2). The significant increase in monocyte CD163 index in both “on-pump” and “off-pump” patients compared to the baseline expression was found only at the 1st and 3rd postoperative days (*P* < .01).

**Figure 3:**
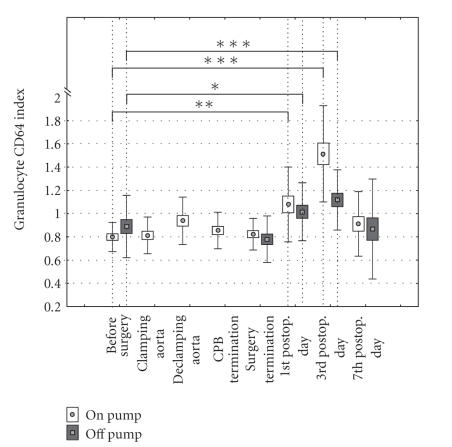
CD64 molecule expression on granulocytes in “on-pump” and “off-pump” patients.

The granulocyte CD64 index was significantly increased both in “on-pump” and “off-pump” patients at the 1st and 3rd postoperative days. Statistically significant differences of granulocyte CD64 indexes between “on-pump” and “off-pump” were not reached (*P* < .195) (Figure 3). The original and pathophysiologically very important observation in our results is that the maximum of CD64 expression on monocytes (1st postoperative day) precedes the maximum of CD64 expression on granulocytes (3rd postoperative day). The granulocyte CD64 expression returned to baseline or normal levels by the 7th postoperative day.

## 7. DISCUSSION

A long-term sustained effort is devoted to the better understanding of inflammatory reaction which is inseparably linked to every cardiac surgical operation. Numerous humoral and cell-mediated parameters of immune system have been studied to identify markers describing the development of such inflammatory reaction. The ultimate goal of such studies is to identify those patients who are at substantial risk for development of overwhelming systemic inflammation (SIRS), a condition of complex physiology that might severely compromise the outcome of surgery or even lead to death. Many studies conducted in the last few years have shown reduced inflammation in patients operated on by the “off-pump” technique compared to “on-pump” surgery [9]. On the other hand, any definitive proof in favor of “off-pump” surgery in terms of reduced long-term mortality compared to its “on-pump” counterpart has not been reported. The very trauma of surgery seems to be more relevant in initiating SIRS rather than cardiopulmonary bypass itself, the latter adding a CPB specific fraction on top of unfavorable events [10].

The objective of this study was to compare the degree of activation of innate immunity cells between “on-pump” and “off-pump” patients. The novel diagnostic Leuko64 kit was used by us to dissect such differences. This kit enables quantitative determination of CD64/CD163 molecule expression on immune cells by flow cytometry.

Fc*γ*R1 receptor (CD64) is constitutively expressed on macrophages, monocytes, and eosinophils, but its expression is negligible on resting neutrophils. Neutrophil CD64 molecule expression is one of many activation-related surface receptors changes manifested during the normal innate immunity response. Microbial cell wall components such as LPS, complement split products, and cytokines (IFN*γ*, TNF*α*) are some of the activators [3]. In contrast to numerous other neutrophilic activation surface antigens, such as CD11b/CD18, CD14, and CD16 with large storage intracellular pools, CD64 has limited intracellular storage, but de novo synthesis can be induced in the presence of proinflammatory conditions. In comparison with former neutrophilic activation markers, CD64 expression on neutrophils is thus much less affected by stimuli of degranulation to which these cells are extensively exposed both during cardiac surgical operation and during blood sample processing [5]. Thus CD64 molecule appears uniquely suited as a surrogate marker of neutrophil activation or systemic acute inflammatory response as its expression starts from less than 2000 sites per cell and becomes upregulated in a graded fashion depending upon intensity of stimulation by cytokines [4].

Clinical usefulness of CD64 determination has been proven in differential diagnosis of sepsis of bacterial origin. Changes in the expression of CD64 on circulating leukocytes in patients undergoing cardiothoracic surgery were also reported [11]. Some of these previous works are suffering from the lack of precise quantitation since CD64 positivity was for example expressed as mean fluorescence intensity [11, 12]. This means of quantitation is valid on a single platform for day to day comparison, but lacks standardization necessary for a routine clinical laboratory test, whereas the Leuko64 kit approach does allow for interlaboratory comparisons. Application of a new standardized diagnostic kit in our study overcomes above mentioned limitations. Using calibration fluorescence beads and specialized software, CD64/CD163 density is expressed as index positivity separately for granulocytes and monocytes. As expected, CD64 positivity on monocytes is one log higher in comparison with granulocytes. A significant increase in both monocytes and granulocytes CD64 expressions has been found on the 1st and 3rd postoperative days. No significant differences between “on-pump” and “off-pump” patients have been recognized. Whereas CD64 expression on monocytes is nearly identical for both “on-pump” and “off-pump” patients, there is a tendency for higher granulocyte CD64 index on the 3rd postoperative day in “on-pump” patients. This likely reflects the greater sensitivity to cytokine effects of granulocytes compared to monocytes. Our results are in contrast to the work of Stefanou et al. [13] who did not report any induction of CD64 expression on monocytes of cardiac surgical patients post CPB. However, only 10 patients were enrolled to their study with some differences in CPB compared to our patients. Furthermore, our attempt using Leuko64 kit ensures better standardisation of method in comparison with previous work, where positivity was expressed as a simple MFI.

The neutrophils CD64 index is designed so that normal inactivated cells yield value of <1.00 and blood samples from individuals with documented sepsis or SIRS typically show values of >1.50. In our patients, the perioperative and postoperative period was uneventfull with the only one exception, the patient, who will be discussed below. It is our original observation that the maximum of CD64 expression on monocytes of our cardiac surgical patients has already been reached on the 1st postoperative day in contrast to granulocytes in which the maximum of CD64 expression has been postponed to the 3rd postoperative day. This observation implies that Fc*γ*RI expression on monocytes is upregulated by a different cellular mechanism very early during operation by the exposition of monocytes to various danger patterns which are raised during operation. The upregulation of Fc*γ*RI on granulocytes is a secondary event mediated by their exposition to proinflammatory cytokines and mediators formed by monocytes-macrophages.

The Fc*γ*RI receptor (CD64) on white blood cells integrate responses involving both the innate and acquired immune systems and are very important for effective phagocytosis of bacteria and immune complexes. A new, intrinsic role for this receptor has recently been proposed by Devaraj et al. who have described the participation of Fc*γ*R on internalization of C-reactive protein by endothelial cells, with subsequent release of chemoattractive proinflammatory IL-8, decrease of eNOS, and increased ICAM-1 and VCAM-1 adhesion molecules [14]. In conclusion, Fc*γ*RI reveals proinflammatory activities in general.

Proinflammatory pathways have to be tightly counterbalanced by numerous anti-inflammatory processes, that is production of anti-inflammatory cytokines such as IL-10 [15], to maintain protective physiological level of inflammatory response in cardiac surgical patients. Another approach to calm down inflammatory reaction is removing of pleiotropic proinflammatory species by the action of so-called scavenger receptors which are differentially expressed on cells of both immune and nonimmune origins [6]. Unique characteristics amongst these scavenger receptors reveal hemoglobin scavenger receptor (CD163). CD163 scavenger receptors represent a highly efficient system to remove potentially toxic and proinflammatory hemoglobin from the circulation and local sites of inflammation [16]. Cardiac surgical patient are extensively exposed to large amounts of free heme/hemeproteins due to intravascular haemolysis and tissue damage. There is accumulating evidence that an excess of free heme can cause cell damage and tissue injury. Heme catalyzes the formation of reactive oxygen species (ROS), resulting in oxidative stress. Because the low-molecular weight iron chelated heme is lipophilic, it can easily intercalate in the membrane and impair lipid bilayers and organelles, such as mitochondria and nuclei, and destabilize cytoskeleton [17]. Several defense mechanisms against free heme-mediated oxidative stress and inflammation exist. They consist of intra- (e.g., heme oxygenase-2 and heme oxygenase-3) and extracellular (e.g., hemopexin, albumin) scavengers, antioxidative enzymes, and heme oxygenase-1 during hemolysis.

Intravascular free hemoglobin is captured by plasma scavenger protein haptoglobin. It is generally accepted that stable hemoglobin-haptoglobin complexes are subsequently delivered to the reticuloendothelial system by CD163 receptor-mediated endocytosis [16]. Any free vascular heme is bound to the plasma protein hemopexin or albumin, which transport it to the liver for degradation in reticuloendothelial system. However, when large amounts of free heme proteins or heme (locally) accumulate, like in a blood clot or after vascular deposition, the scavengers get overwhelmed or are unable to reach them. This enables heme to exert its damaging effects. Therefore, the amount of free heme must be tightly controlled to maintain homeostasis and avoid pathological conditions.

The monocyte CD163 index has been found to be between 5 000 and even 40 000 in our study. There was a sharp increase of monocyte CD163 in the postoperative period reaching statistically highly significant maximum (*P* < .001) on the 1st postoperative day for both “on-pump” and “off-pump” patients followed by decrease to the baseline preoperative levels. Furthermore, the patients’ monocyte CD163 index is significantly higher (*P* < .001) in “on-pump” patient group on the 1st postoperative day. Reports regarding monocyte CD163 expression in cardiac surgical patients are very sparse. In a study by Goldstein et al. [18], a significant increase in the monocyte CD163 expression on the 1st postoperative day was found. This is in accord with results of our study. But there were several limitations in a previous study. CD163 molecule was expressed as mean fluorescence index (MFI) in comparison with our study in which standardized quantitative approach using calibration fluorescence beads was exploited yielded much more relevant data. The changes in the entire perioperative period and during postoperative period up to the 7th postoperative day have been followed by us in comparison with a previous study.

The link of CD163 hemoglobin scavenger receptor to anti-inflammatory phenomena in cardiac surgical patients has been proven by Philippidis et al. [19]. In their study, elevated expression of CD163 on circulating monocytes during the resolution phase of the systemic inflammatory response to cardiopulmonary bypass surgery was reported. Furthermore, binding of hemoglobin-haptoglobin complexes to CD163 expressing monocytes elicited potent interleukin-10 secretion. It was reported by Goldstein et al. [18] that an increase in the monocyte CD163 expression was 14 times higher in their “on-pump” patients who were treated by bolus administration of metylprednisolone perioperatively in comparison with untreated patients. Such therapeutical intervention was not performed in any our patients. It seems unlikely that higher monocyte CD163 expression in our “on-pump” patients was elicited by metylprednisolone which is a regular component of CPB fluids used at our department. More pronounced proinflammatory stimuli raised during “on-pump” surgery are more relevant. There will be the unique chance to dissect between the influence of corticosteroids present in CPB fluid and other variables involved in “on-pump” surgery. Currently, CPB protocol used at our department has been changed and methylprednisolone has been ommited. CD163 is rapidly shed from the surface of monocytes when activated by LPS or phorbol esters. In addition, also cross-linking of Fc*γ*R triggers shedding of CD163 [20]. This phenomenon is very likely counterbalanced by the fact that such shedding is followed by upregulation of this hemoglobin scavenger receptor, as was shown for metaloproteinases-mediated CD163 shedding [21]. This is probably true for our cardiac surgical patients. Very recently, it was proven by Weaver et al. [22] that CD163 shedding is also induced via stimulation with TLR-4, TLR-2, and TLR-5.

Initially included in this study was a single patient, male, 73 years old who was excluded from our study due to his death on the 2nd postoperative day. This patient underwent uncomplicated cardiac surgery using cardiopulmonary bypass. He developed acute diaphragmatic myocardial infarction two hours after finishing surgery. Patient was reoperated and reanastomosis was performed. In spite of this effort, cardiogenic and subsequent hemorrhagic shock was developed ultimating to death next day in the morning. Body temperature was below $36^{\circ }$C so that infection is unlikely.

The expression of both CD64 and CD163 molecules before surgery and up to the end of surgery was comparable with other patients. Granulocyte CD64 index reached 1.72 value which is significant for the development of SIRS [23]. However, such values were found in additional four patients without any impact on their postoperative course. Remarkable is the fact that monocyte CD163 index in this patient was the highest between our patients and was approximately two times higher in comparison with all our patients investigated. It could be concluded from this our anecdotal observation that the increase in CD163 monocyte positivity could be the marker with predictive value of worse outcome in cardiac surgical patients.

In conclusion, using standardized quantitative methods we revealed substantial dynamics in the expression of the activation marker Fc*γ*RI and scavenger receptor CD163 in both “on-pump” and “off-pump” cardiac surgical patients during operation and in the postoperative period. The maximum in the expression of CD64 on monocytes in postoperative period precedes the maximum in the expression of this molecule on granulocytes by two days.
